# Controls and constrains of the membrane disrupting action of Aurein 1.2

**DOI:** 10.1038/srep16378

**Published:** 2015-11-17

**Authors:** Mahdi Shahmiri, Marta Enciso, Adam Mechler

**Affiliations:** 1 Department of Chemistry and Physics, La Trobe Institute for Molecular Science, La Trobe University, Bundoora Vic 3086, Australia

## Abstract

Aurein 1.2 is a 13 residue antimicrobial peptide secreted by the Australian tree frog *Litoria Aurea*. It is a surface-acting membrane disrupting peptide that permeabilizes bacterial membranes *via* the carpet mechanism; the molecular details of this process are mostly unknown. Here the mechanism of action of Aurein 1.2 was investigated with an emphasis on the role of membrane charge and C-terminal amidation of the peptide. Using quartz crystal microbalance (QCM) fingerprinting it was found that the membrane charge correlates with membrane affinity of the peptide, however the binding and the membrane disrupting processes are not charge driven; increased membrane charge reduces the membrane disrupting activity. Coarse grain simulations revealed that phenylalanine residues act as membrane anchors. Accordingly Aurein 1.2 has the ability to bind to any membrane. Furthermore, bundling precludes membrane disruption in case of wild type peptides, while non C-terminal amidated peptides form random aggregates leading to detachment from the membrane. Hence C-terminal amidation is crucial for Aurein 1.2 action. Our results suggest that Aurein 1.2 acts *via* aggregation driven membrane penetration. The concomitant change in the tension of the outer leaflet imposes a spontaneous curvature on the membrane, leading to disintegration.

The emergence of antibiotic resistant strains of common pathogens is an increasing concern worldwide, prompting a quest for novel therapeutic approaches^1^. Among the alternatives to traditional antibiotics, antimicrobial peptides (AMPs) attract growing interest^2^,^3^. AMPs form a vital part of the innate immune system in organisms including plants, amphibians, insects and mammals^4^,^5^. A class of these antimicrobial peptides kill pathogens by permeabilizing their plasma membrane *via* a specific, but not receptor-mediated mechanism^4^,^6^,^7^. This mode of action offers a promising alternative to existing therapeutic agents to overcome the resistance problem^8^. It is known that the lytic effect is defined by the peptide sequence and the characteristic lipid composition of the membrane; the AMPs in their host organisms are specific and selective to pathogenic membranes^9^. Unfortunately, most wild type peptides originate from non-mammalian hosts and thus also disrupt mammalian cells; however, peptides designed for pharmaceutical purposes may provide a viable treatment option^10^.

Designing AMPs for pharmacological activity assumes the knowledge of their molecular mechanism of action. AMPs are usually assigned into two main categories based on their mode of membrane disruption: surface acting peptides and transmembrane pore formers. The distinction is largely empirical with a weak correlation to the size of the peptides. AMPs greater than 20 amino acid residues are long enough to span a lipid membrane in an α-helical conformation^11^,^12^ forming either barrel-stave^13^ or toroidal pores^14^, while shorter peptides that are unable to span the membrane are more likely to act *via* the “carpet” mechanism^13^. In the phenomenological model of the carpet action, peptides first bind to the surface of the target membrane and cover it in a carpet-like manner, then, after a threshold concentration has been reached, the peptides cause a sudden breakdown in the membrane integrity^15^. The threshold concentration of disruption depends on the type of the target membrane and can occur after either the entire surface of the membrane or local areas are saturated with peptide^16^. Importantly, the peptides remain tightly bound to the membrane interface throughout the mechanism^13^,^17^,^18^. However, not much is known about the mechanistic pathway leading from membrane attachment to membrane disintegration.

The specificity of AMPs to pathogens is frequently explained with their positive charge^19^,^20^. Consistently, cationic peptides are reported to be more active against Gram positive bacteria^21^ while the complex protective structures including a lipopolysaccharide layer and outer membrane reduce or eliminate activity against Gram-negative bacteria^22^. Charge effects have been studied extensively for transmembrane peptides and a clear correlation is recognized between the AMP charge and antimicrobial activity^23^,^24^,^25^. However a higher charge is not always advantageous as it interferes with structuring^26^ or increases haemolytic activity parallel to an increased antibacterial efficiency^27^. In case of the carpet action, it is assumed that at least the initial membrane binding step is electrostatically driven since AMPs that follow the carpet mechanism typically carry a high positive charge^16^. However the exact role of peptide charge in the carpet mechanism is not known.

Structural factors also play a role in the activity of antimicrobial peptides^28^. The percentage α-helicity has a strong correlation to antimicrobial activity^29^,^30^. C-terminal amidation is a structural feature present in most of wild type peptides and it is believed to contribute to enhancing antimicrobial efficacy^31^,^32^ through stabilizing the α-helix^33^,^34^. Since C-terminal amidated peptides have a higher positive charge than those with a free C-terminus, it is also possible that the charge is the causal factor in their higher activity, although charge alone is not sufficient to describe the differences^28^. Other authors suggested that C-terminal amidation can prevent the enzymatic degradation of antimicrobial peptides^35^. The specific role of the C-terminal amidation on the molecular mechanism, in particular of the carpet action of AMP activity remains unclear.

Aurein 1.2 is a 13-amino acid peptide (GLFDIIKKIAESF-NH_2_) secreted by the Australian green and golden bell frog Litoria aurea and by the related Australian southern bell frog Litoria reinforms^36^ and is the smallest AMP secreted by vertebrates. Given its relative simplicity Aurein 1.2 is a suitable model to study the mechanism of the carpet action of antibacterial activity. Aurein 1.2 shows specificity to charged lipids which is believed to be a targeting mechanism to bacteria^37^. Aurein 1.2 is unstructured in aqueous solution, adopting an α-helical geometry when incorporated into lipid membranes^38^,^39^. In the membrane environment it has an amphipathic α-helix with well-defined hydrophilic and hydrophobic regions^36^ that is more commonly found in pore forming peptides^12^,^40^.

There are conflicting reports in the literature about the membrane disrupting threshold of Aurein 1.2. In charged liposomes Aurein 1.2 causes dye leakage from 1–4 μM in a concentration-dependent manner, achieving complete membrane lysis at higher concentrations^41^,^42^, whereas quartz crystal microbalance (QCM) experiments suggest complete solubilization of charged supported bilayers at all concentrations^43^. In contrast, other biophysical studies suggest that there is little membrane disordering at concentrations below 7–10 μM while at higher concentrations Aurein 1.2 can disrupt both zwitterionic DMPC and charged DMPC/DMPG membranes, albeit to a different degree^37^ or with a different affinity^29^,^44^. Reported threshold values vary with the measurement technique used and hence the definition of threshold, as well as the phase of the membrane; however, thresholdless disruption of charged membranes has been observed in both fluid and gel phase^41^,^43^.

Importantly, the action of Aurein 1.2 is sensitive to local structure of the membrane^45^,^46^, hence the role of the support chemistry and surface morphology might be responsible for the documented variations in the biophysical measurements. Phospholipids form tightly bound, rigid lamellar bilayers on metal oxide surfaces that exhibit only negligible dissipation in QCM measurements^47^,^48^, whereas lamellar membranes of the same composition are partially suspended and highly dissipative on carboxylic acid-functionalized surfaces^49^. Hence the same phospholipid bilayer can exhibit substantially different viscoelastic properties if deposited onto different substrates^49^. If the membrane is not a homogeneous single bilayer, lamellar stacking effects^50^ and/or intact liposomes also influence viscoelastic properties; the former are frequent on DMPC deposits whereas the latter is typical of DMPC:DMPG membranes^51^. Therefore, using a membrane of well controlled properties is key to study the mechanism of action in isolation from the aforementioned variables.

In this current work, highly biomimetic partially suspended membranes were used^49^. Anionic and zwitterionic *single bilayer* biomimetic membranes were exposed to different concentrations of wild type and non-amidated Aurein 1.2 on a QCM sensor surface. The methodology is based on previous works using dual beam polarization interferometry and QCM to fingerprint AMP membrane disruption^29^,^30^,^44^. The steps of the molecular mechanism as well as the effect of cholesterol content, increasing membrane charge and C-terminal amidation have been studied. DMPC with or without cholesterol is a widely used model for neutral membranes in the literature of AMP membrane interactions^9^,^37^ while DMPC/DMPG lipid mixtures are frequently used to model charged membranes^52^, hence these lipid mixtures were employed in this study. The mechanism was further investigated using molecular dynamics simulations.

## Results and Discussion

**Figure 1:**
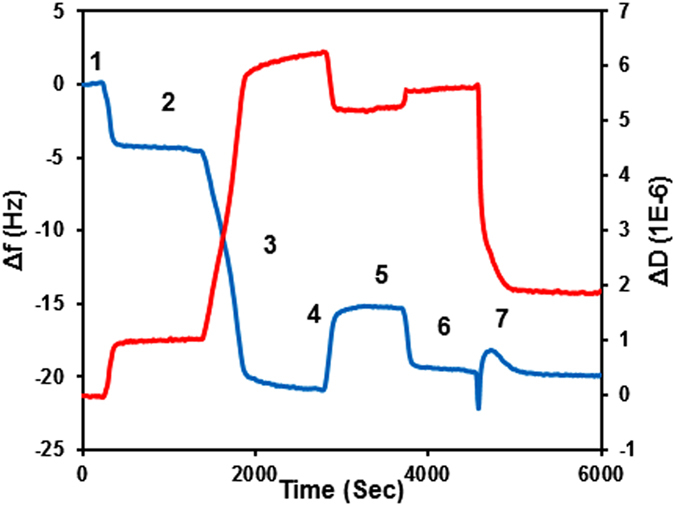
Typical sensogram of a QCM experiment (DMPC, 7 μM Aurein 1.2). (**1**) Initial water baseline; (**2,4,6**) PBS buffer; (**5**) buffer with low salt concentration (30 mM), (**3**) lipid deposition curve, and (**7**) peptide effect. The difference in frequency between baseline 2 and 6 was used to calculate a total amount of lipid on the chip surface. Mass loss between 4 and 6 indicates removal of unopened liposomes and/or secondary bilayer patches from the membrane surface.

The interactions of wild type Aurein 1.2 and non-C-terminal amidated Aurein 1.2 (Aurein 1.2 -COOH) with supported phospholipid bilayers of DMPC, DMPC/DMPG (4:1), DMPC/Cholesterol (9:1), and DMPC/DMPG (3:2) compositions, respectively, were recorded using QCM. In a typical experiment (Fig. 1) after obtaining a stable baseline in water (1) and assay buffer (2) liposome suspension was introduced into the chamber (3) leading to negative frequency shift, indicating lipid deposition (4). In all measurements the deposit was then rinsed with a “low salt” buffer (30 mM NaCl concentration) to remove unopened liposomes from the surface by osmotic stress (Fig. 1(5)). After returning the assay buffer to the measurement chamber (6) the frequency change was at ~14 Hz and dissipation at ~3 × 10^−6^, which is consistent with a single bilayer deposit^49^,^51^. In all experiments peptide solutions were only introduced once the presence of a single bilayer membrane was confirmed (Fig. 1(7)).

**Figure 2:**
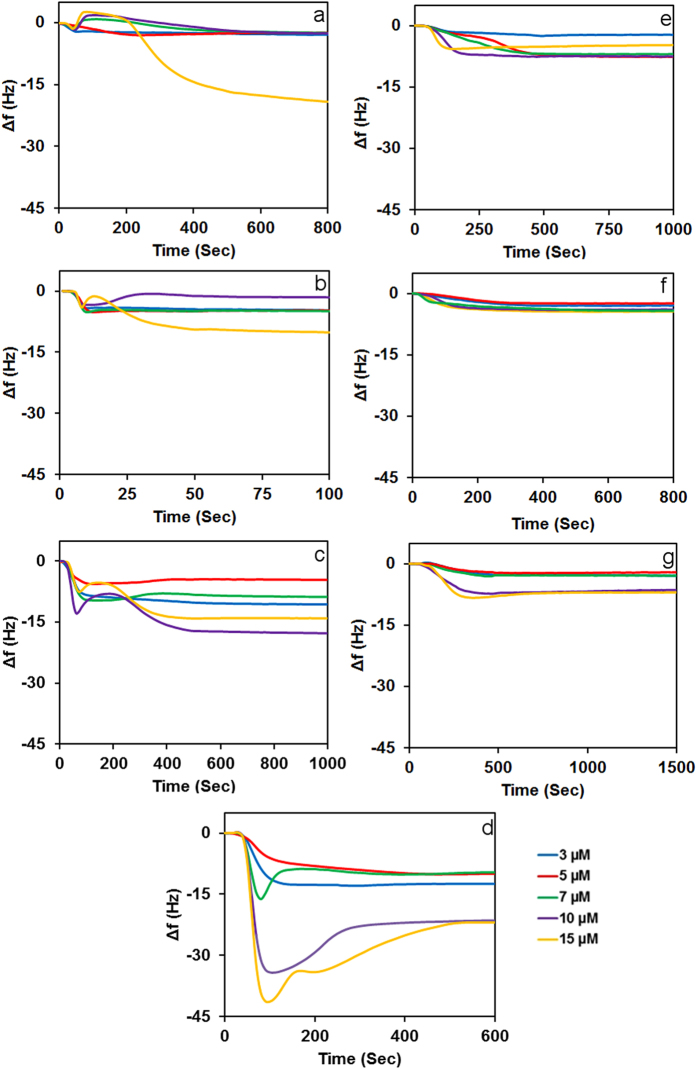
Frequency changes of the interaction of (3, 5, 7, 10, and 15 μM) Aurein 1.2 (wild type) with (a) neat DMPC, (b) DMPC/Cholesterol, (c) DMPC/DMPG (4:1), (d) DMPC/DMPG (3:2), and Aurein 1.2-COOH with (e) neat DMPC, (f) DMPC/Cholesterol, (g) DMPC/DMPG (4:1).

Figure 2 shows the frequency change (Δf) vs time (t) sensograms for wild type Aurein 1.2 and Aurein 1.2-COOH with various membranes. In the case of 3 μM peptide concentration (Fig. 2(a)) there is only a small negative frequency change. However at 5 μM after an initial drop there is a little increase in *f*. Increasing the concentration further to the 7 and 10 μM resulted in a similar but more pronounced trend with a frequency increase to values above the initial *f*. At 15 μM this trend is followed by a steep frequency drop. The initial decrease in *f* that is observed at all concentrations is consistent with peptide binding to the membrane surface. Above 5 μM concentration, the initial negative trend is followed by a small increase in frequency that is followed by a second, slow negative trend for all concentrations, exhibiting a “wave” pattern that accelerates over time at 15 μM.

Addition of 10% cholesterol to the DMPC membrane (Fig. 2(b)) changed this interaction pattern. There is only binding in the case of 3 and 5 μM concentrations, whereas at 7 μM after the initial binding there is a slight increase in *f*. Increasing the concentration further to 10 μM enhances the second process. Upon increasing concentration to 15 μM a third process, a substantial negative frequency change, is observed, similar to the pattern seen for neat DMPC with 15 μM Aurein 1.2.

Addition of 20% DMPG to the DMPC membrane (Fig. 2(c)) resulted in very different interaction characteristics. At 3 μM there is only binding. Over 5 μM concentration there is an increase in Δ*f* after the initial binding. At 10 μM concentration a third trend also becomes visible: an additional negative change to produce a “wave” pattern. This result is consistent with earlier QCM work on gel phase membranes^29^,^44^ but different from the behaviour of Aurein 1.2 with fluid membranes where even small concentrations of Aurein 1.2 lead to complete removal of DMPC/DMPG (4:1) from the surface^43^. However, complete disintegration of gel phase membranes by Aurein 1.2 was also observed in dye leakage studies^41^, hence membrane phase might not be a determining factor in the observed differences. It was established before that Aurein 1.2 action is highly sensitive to the local curvature^45^; hence the different result in this study is likely due to the better control of membrane morphology, ensuring the absence of intact small liposomes or small secondary bilayer patches, both of which offer high local curvatures for preferential Aurein 1.2 attack.

Cationic peptides have shown a stronger binding affinity to anionic lipids^53^. Therefore, electrostatic interactions are thought to be the main driving force in the carpet mechanism, with a potential role played by hydrophobicity as well^8^,^15^,^54^. Accordingly, increasing the charged lipid content of a membrane should lead to higher peptide activity. To test this assumption a mixture of DMPC/DMPG (3:2) was exposed to Aurein 1.2 (Fig. 2(d.)) At 3 and 5 μM substantially more peptide is bound to the membrane than in the case of DMPC:DMPG (4:1) membrane, as expected if charge interaction plays a role; however even at higher concentrations the “wave” observed for the DMPC:DMPG (4:1) interaction is largely absent.

To gain better insight into the effect of C-terminal amidation on the mechanism of action of Aurein 1.2 on membranes, Aurein 1.2-COOH was also studied (Fig. 2(e–g)). In all cases, decreasing frequency indicates that Aurein 1.2-COOH is able to bind to the membrane without significant disruption although at 15 μM on neat DMPC and DMPC:DMPG (4:1) slightly lower frequency change indicates the onset of a weak secondary process. Hence our experimental results show that C-terminal amidation has a profound effect on the interaction mechanism, which is consistent with recent literature results on other aurein peptides^55^.

### Fingerprinting analysis

The differences in the membrane interactions of Aurein 1.2 and Aurein1.2-COOH can be analyzed through changes in the membrane viscoelasticity. Hence the mechanism of action of these peptides was further explored by using the mechanistic “fingerprints” formed when plotting the QCM dissipation change against the frequency change (ΔD vs Δ*f*). Such fingerprints of a mechanism are used primarily to identify stages or sub-processes that exhibit different viscoelastic character. The trendline [–f, +D] reveals the formation of a thin viscous layer, that is, mass uptake; conversely a trendline towards [+f, –D] usually indicates mass loss^56^. For trendlines in [–f, –D] and [+f, +D] directions, the change predominantly occurs in viscoelastic properties, that is, distribution, density or mixing of the constituents of the deposit; these are referred to as structural changes^29^,^57^. These viscoelastic processes can be *qualitatively* assigned if informed by other experimental methods, computational results or by assessing possible mechanistic pathways.

**Figure 3:**
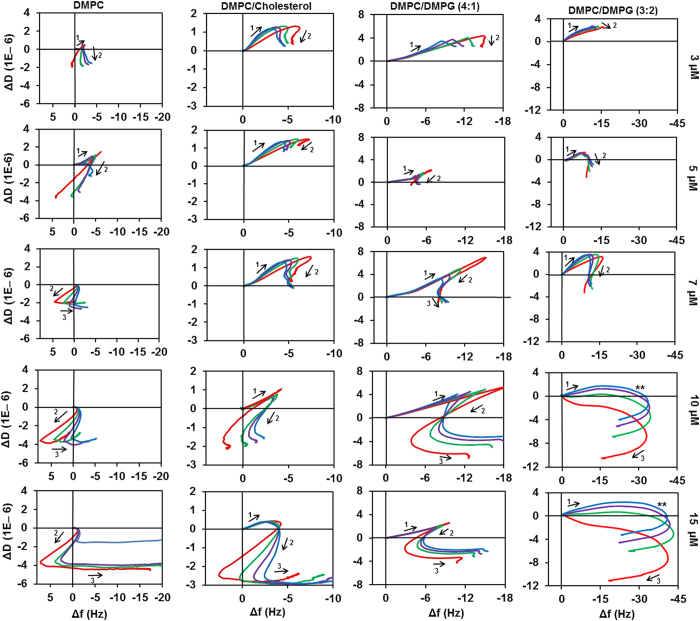
Plots of ΔD-Δf of wild type Aurein 1.2 with different membranes at varied concentrations (3, 5, 7, 10, and 15 μM). The effect is shown for the third (red), fifth (green), seventh (purple), and ninth (blue) harmonic of the fundamental frequency of the quartz chip.

Figure 3 shows the effect of wild type Aurein 1.2 on the four different membranes. Changes in the direction of the curves reveal discrete stages of the mechanism. Thus, Aurein 1.2 interaction shows a concentration-dependent three stage mechanism with each of the four lipid mixtures studied. However there are marked differences between the characters of these stages in the different lipid mixtures.

Stage 1 in all cases points to [−f, +D] direction, indicating homogeneous mass uptake. The amount of binding differs by membrane type: in DMPC it is ~ − 4 Hz but the stage is only discernible at low concentrations, suggesting two competing equilibria between solution phase and surface bound peptide as well as e.g. monomeric and aggregated form on the membrane. The existence of peptide adsorption equilibria and the ability of the membrane to recover from low concentration exposure of AMPs has been described before^30^. Addition of cholesterol preserves stage 1 for higher concentrations. Importantly, the maximum frequency change at the end of stage 1 does not depend on peptide concentration. This implies that it is related to a certain degree of surface coverage, suggesting saturation. However, charged lipid increases the maximum binding in stage one; on DMPC:DMPG (4:1) membrane the maximum value is ~ − 12 Hz at higher concentrations of Aurein 1.2, with a maximum dissipation of ~5–7 a.u., after which there is a sharp change to a [+f, –D] trendline in stage 2. In stark contrast, mass uptake continues to −30–40 Hz for DMPC:DMPG (3:2). However, after reaching maximum value at ~12 Hz (approximately at the same values as in case of DMPC:DMPG (4:1)), the dissipation trend becomes negative (**), indicating that further mass uptake restrains membrane mobility, the primary cause of dissipation^49^. It is feasible to assume that this trendline (**) is a separate stage for this lipid mixture, albeit it is not as distinct as in case of DMPC:DMPG (4:1). Hence, increasing membrane charge delays the onset of mass loss, that is, it is *hindering* membrane disruption.

Stage 2 is highly lipid-dependent. In case of neat DMPC this is initially a [+f, −D] trend (Fig. 3 DMPC 3 μM) that moves towards [−f, −D] direction at higher concentrations, with a substantial spread of the harmonics. As described previously^29^,^58^, this indicates a surface process, which is primarily a structural change; a concomitant decrease to water coupling accounts for the negative frequency change^29^. In case of DMPC with 10% cholesterol content, a similar trend is observed that is shifted to higher concentrations. Importantly, stage 2 is different for charged membranes and has a remarkable sensitivity to DMPG lipid content. In case of 20% DMPG, the trend is [−f,−D] with mostly overlapping harmonics of the crystal resonance at low concentrations, which starts to show spreading at 10 μM. In case of 40% DMPG content, the second stage is unclear, as discussed above; (**) is primarily a [−f, −D] trend with lessening dissipation change at higher concentrations. Mass uptake itself can only cause a positive dissipation change. Yet the continuing negative frequency change suggests that more peptide is binding to the membrane surface. Hence the only possible interpretation is the overlapping of mass uptake [−f, +D] and a parallel structural change [−f, −D]. This is consistent with further peptide binding to the membrane that was made accessible by aggregation of peptide. Therefore stage 2 reflects the key differences between the way Aurein 1.2 interacts with membranes.

Stage 3 is an abrupt change in the trend line, which has been registered for all lipid mixtures over a threshold concentration. This observation has not been reported in the literature before. It is likely related to the precondition of suspended single bilayer membrane coverage of the chip surfaces, which was not ensured in previous studies. As we described above, the concentration threshold depends on the membrane composition: for DMPC, it is at 7 μM; for DMPC:Cholesterol (9:1) at 15 μM whereas for both DMPG containing mixtures it is at 10 μM. However, in case of 40% DMPG content the trend line is weakly [+f, −D], the opposite of the trend observed for all other mixtures (where it is a nearly pure negative frequency change).

The near zero dissipation change that accompanies the large negative frequency changes suggests that a tightly coupled rigid layer is forming on the surface. Apparent mass gain without any change in dissipation is an unexpected feature since previous studies suggest membrane dissolution which would result in continuing [+f, −D] mass removal to frequency change values above zero that would indicate the removal of the membrane^29^,^43^,^57^. Hence, based on the well documented membrane disrupting ability of Aurein 1.2 at 10–20 μM concentrations, stage 3 has to be related to the disintegration of the membrane. One possible explanation is that the dissolution of the membrane exposes the MPA surface to further peptide binding. However, control experiments with 10 μM Aurein 1.2 on MPA modified sensor chip surface recorded only ~1 Hz frequency change, hence this explanation can be ruled out. The only other possibility is a fundamental re-packaging of the peptide and lipid into disruption products such as mixed micelles that nevertheless remain on the chip surface due to the strong attachment to the MPA carboxyl groups^57^. That also suggests that the final step of membrane disintegration allows the incorporation of more peptide into the disruption products from the solution phase; consistently the largest frequency changes in stage 3 are observed at the highest peptide concentrations. Therefore, while this behaviour is markedly different from the material removal observed for multilayer membranes, it is not a contradiction in terms of the mechanism.

### Coarse grain simulations

**Figure 4:**
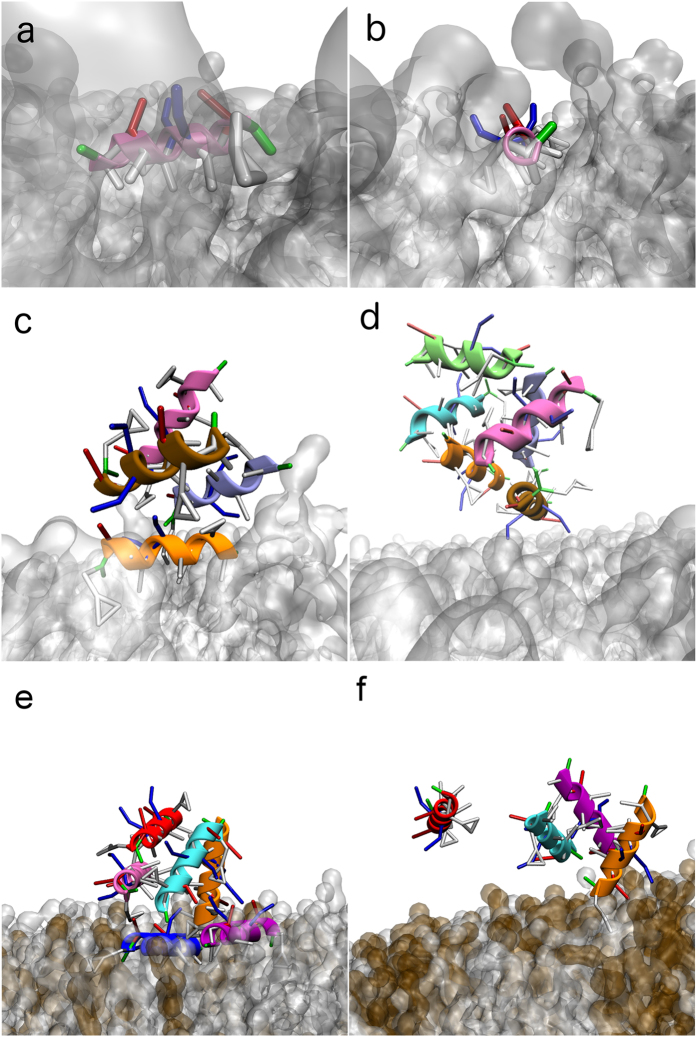
Representative snapshots from the coarse grain simulations. Amino acids are coloured according to their chemical nature (white, hydrophobic; green, hydrophilic; red, negatively charged; blue, positively charged). The α-helical backbone is also shown, each peptide in a different colour. (**a**) Side view of the membrane inserted peptide. Note that the “triangular” side chains correspond to the phenylalanine rings in a MARTINI representation. (**b**) Front view of a membrane inserted peptide. (**c**) Interaction of wild type peptides with DMPC. (**d**) Interaction of non-amidated peptides with DMPC. (**e**) interaction of wild type peptides with DMPC:DMPG (4:1) (DMPG in brown). (**f**) interaction of wild type peptides with DMPC:DMPG (3:2).

Single-peptide simulations show that both amidated and non-amidated Aurein 1.2 spontaneously bind to a DMPC lipid membrane. A movie of a representative simulation is available as supplementary material. Phenylalanines 3 and 13 are the key anchors, with their rings (in Fig. 4 these appear as triangles in MARTINI representation) interacting with the apolar part of the lipid. Due to the helical conformation of the peptide, the two phenylalanines have different insertion geometries: Phe3 is nearly parallel to the surface (at an angle of 20 degrees) whereas Phe13 is more perpendicular (45 degrees). This orientation is observed in both wild type and non-amidated peptides (Fig. 4(a,b)). The peptides insert about 1.75 nm inside the membrane, with the amphiphilic helix resting at the boundary between the headgroup zone and the hydrophobic core.

Placing six peptides in the box simulations have been carried out for the same four types of membranes that were used in the experiments. In all cases, aggregation is observed over time. In the case of the wild type peptides and DMPC membrane, aggregates are formed *via* hydrophobic interactions (mostly mediated by isoleucine), resulting in a bundle. The aggregated structures remain anchored to the membrane through phenylalanines as described above (Fig. 4 (c)). Non-amidated peptides also aggregate (see Fig. 4(d)), however the aggregates are disordered, they lose the membrane anchors and detach from the membrane, although some aggregates may remain loosely attached to the membrane through polar interactions.

Introducing 10% cholesterol to the DMPC lipid did not change the binding geometry of wild type Aurein 1.2, however the binding affinity was weaker with five peptides remaining in the solution phase (not shown). This implies that cholesterol hinders membrane binding and shifts the peptide activity towards higher concentrations, consistent with experimental observations. Charged lipids had the opposite effect. 20% DMPG lead to strong binding with the same geometry as in case of neat DMPC; bundling of the peptides was also observed on the surface (Fig. 4(e)). Importantly the bundle tended towards vertical orientation compared to the membrane plane. Increasing the membrane charge lead to change of behaviour. For 40% DMPG, the Phe peptide anchoring is weakened, likely due to the dominance of the charge interactions. The peptides assemble into a vertical bundle, with only one Phe residue remaining in the membrane (Fig. 4(f)). This is consistent with the experimentally observed anomalous stage 2 of the interaction; vertical orientation of peptides allows for more binding, while also restraining freedom of motion due to packing constraints that leads to reduced dissipation.

These observations provide microscopic insight into the initial stages of the interaction between Aurein 1.2 and the membrane. Full membrane disruption has not been observed in any case, as it is beyond the time scales accessible with these simulations.

### Membrane charge and Aurein 1.2 binding affinity

It was found that Aurein 1.2 binds to both zwitterionic and negatively charged membranes. However, more peptide bind to the charged lipid and increasing anionic lipid content increases the amount of membrane bound peptide. Initial interaction of cationic AMPs with anionic membrane is generally considered to be purely electrostatic, forming the basis of selectivity to bacteria^20^; indeed it was shown that Aurein 1.2 forms complexes with negatively charged polysaccharides as well^59^. Yet, Aurein 1.2 binding to zwitterionic lipid has been reported before with QCM and SPR measurements^38^,^44^. It is argued that, while electrostatic attraction between the positively charged residues of Aurein 1.2 and negatively charged phosphate moiety of the phosphatidylcholine head group of DMPC is highly probable, in comparison to negatively charged lipids such as DMPG, the overall strength of such interaction will be attenuated through competition with the positively charged choline at the surface of DMPC bilayer^38^. Our observations suggest that Aurein 1.2 binding to DMPC lipids is not negligible and the main difference compared to charged lipids is in the onset of stage 2 of the mechanism. Addition of 10% cholesterol increases peptide binding; it implies that charge interaction is not the only binding mechanism, in line with the observation that Aurein 1.2 disrupts zwitterionic as well as charged lipids, albeit at higher concentrations (vs. ~10 μM for DMPC:DMPG (4:1))^38^. Comparing DMPC:DMPG (4:1) and DMPC:DMPG (3:2), the onset of disruption is shifted to higher concentrations, implying that the excess charge has an inhibitive effect on membrane disruption. Our computer modelling, in agreement with former NMR results, show that the peptide is anchored into the membrane with Phe3 and Phe13 residues, that is, with its hydrophobic side, and not with the charged residues. Hence the higher affinity of Aurein 1.2 to charged membranes is a weak targeting motif; differences in the mechanism of action are not charge related.

The interaction of Aurein 1.2 with DPMC/Cholesterol shows weaker activity than with neat DMPC. Since mammalian cell membranes contain cholesterol whereas bacteria do not, this difference reveals a secondary motif for specificity and selectivity. It has been shown that cholesterol attenuates the insertion of AMPs into membranes^60^. Given that cholesterol modulates the fluidity of membrane^61^, this effect suggests that mechanical membrane properties also play a role in the mechanism of action.

### The role of C-terminal amidation

**Figure 5:**
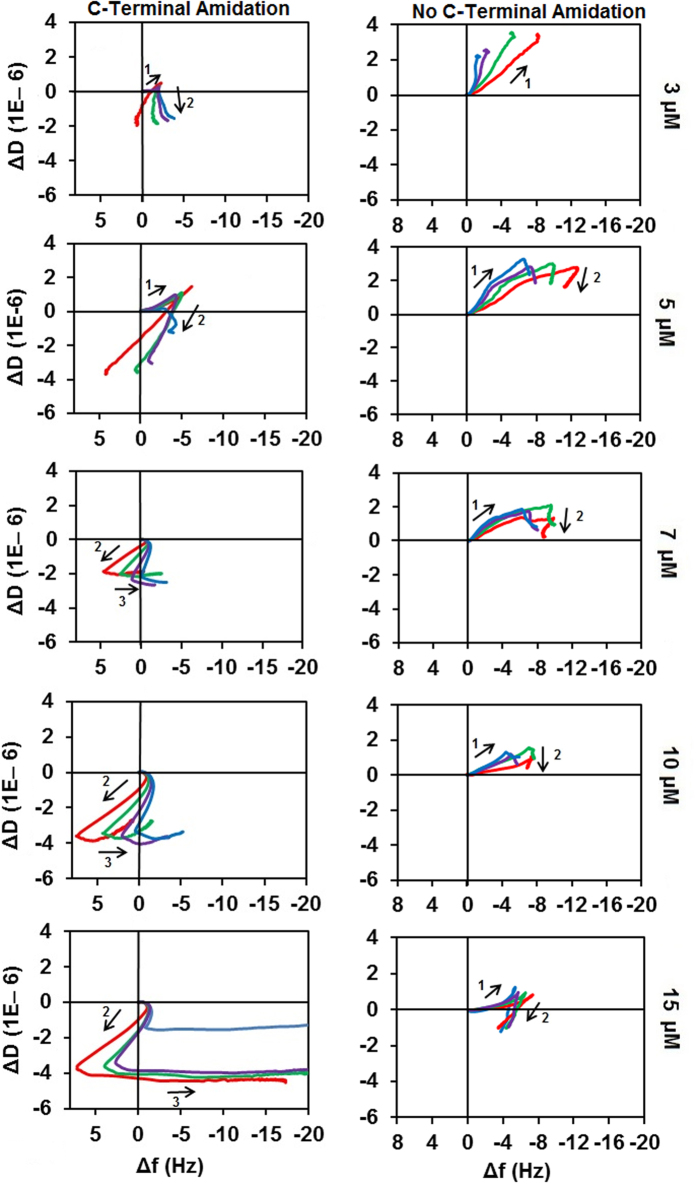
ΔD vs Δf plots of wild type Aurein 1.2 and Aurein 1.2 - COOH interaction with DMPC at different concentrations. Plots are shown for the third (red), fifth (green), seventh (purple), and ninth (blue) harmonic of the fundamental frequency of the sensor chip.

The comparison of ΔD vs Δf plots for neat DMPC exposed to wild type Aurein 1.2 and Aurein 1.2 -COOH is shown in Fig. 5. Even though both peptides bind to the membrane, Aurein 1.2 -COOH did not cause any discernible disruption at any of the concentrations studied. This is consistent with previous results identifying an initial binding phase which is comparable for the amidated and non amidated peptides^28^; this has also been observed in our binding simulations to a DMPC membrane. However, aggregation simulations show that amidation has a key role in further stages, as the wild type peptide remains on the surface whereas the non amidated peptide dissociates from the membrane. Thus the results suggest that C-terminal amidation plays a direct role in membrane disruption by promoting peptide aggregation and potentially aiding membrane penetration by inverting the charge of the C terminus.

### Mechanism of action

**Figure 6:**
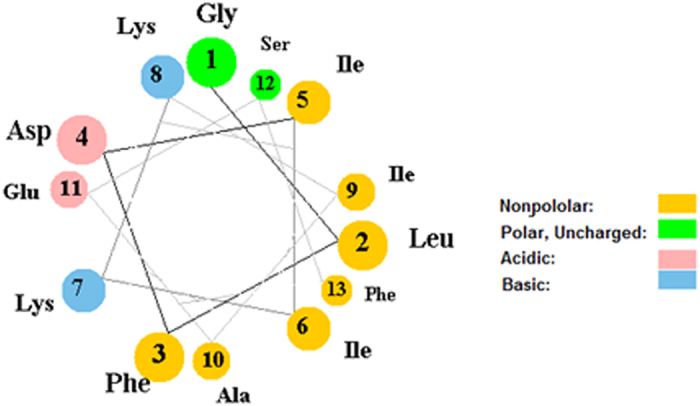
Helical wheel representation of Aurein 1.2.

Rozek *et al*.^36^ calculated the most stable structure of Aurein 1.2 with the hydrophobic residues on one side and the hydrophilic residues on the another side of the helix. The helical wheel representation of Aurein 1.2 is shown in Fig. 6. Such a highly amphiphilic structure is more commonly associated with pore forming peptides, as it leads to hydrophilic or hydrophobic aggregation in helical conformation^13^,^54^. In solution the propensity to aggregate is reduced by weaker α-helicity of the peptide; in the membrane environment however Aurein 1.2 is highly helical^41^, thus the helices can interact as amphiphiles. Consistently, aggregation was revealed by the coarse grain simulations. Yet all evidence suggest that the mechanism of action of Aurein 1.2 is carpet-like^9^,^43^,^57^.

Our simulation results have confirmed the membrane anchor role of phenylalanine residues at 3 and 13 positions that place the amphiphilic helix at the boundary between the headgroup zone and the membrane core. Aurein 1.2 is significantly more active than Aurein 1.1 that lacks Phe13 residue, suggesting that the Phe3 and Phe13 play an important role in the membrane disrupting activity^36^.

Of the carpet action of Aurein 1.2, it is known that the peptide binds to both zwitterionic and anionic membranes^43^, it dissolves the membrane at a certain threshold that is a function of membrane composition^62^, and it remains on the surface of the membrane until the sudden breakdown of membrane integrity^52^. No intermediate stages have been identified thus far between surface attachment and dissolution products. The latter necessarily contain lipid molecules and peptides, and are too small for optical detection, hence believed to be mixed micelles^43^,^62^.

The existence of the threshold suggests that the peptide does not act as a detergent^13^, removing individual lipid molecules from the membrane, but rather as a membrane modulating agent, changing the collective properties of the membrane until it becomes unstable. Three feasible pathways can achieve this. a) If the peptides reside in the lower headgroup area upon membrane binding, a surface coverage can be reached where the lipid molecules are separated so far that the top leaflet integrity breaks down. Disintegration products are likely irregular. b) At a certain surface density the peptides start penetrating the membrane (due to Boltzmann distribution a higher energy inserted state becomes accessible once the surface state is saturated), leading to swelling of the top leaflet of the membrane that introduces a spontaneous curvature; the membrane starts blistering and breaks into spheres. c) Over a certain surface coverage the peptides aggregate into a bundle that favours penetration into the membrane core (hydrophobic side faces outwards), increasing top leaflet pressure, and disintegrating the membrane as in (b). Of these pathways, our results point at (c).

**Figure 7:**
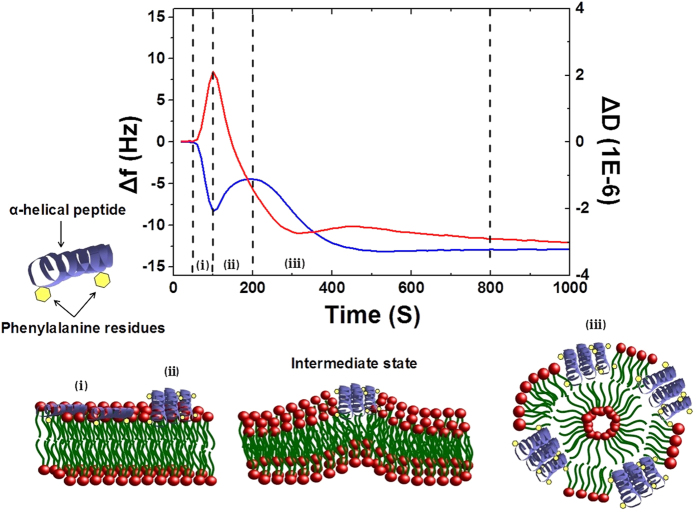
Proposed mechanism of action of Aurein 1.2 Top, frequency (blue) and dissipation (red) sensograms of membrane disruption, with the stages of the process indicated with roman numerals. (**I**) binding; (**II**) aggregation; (**III**) membrane penetration-induced mixed micelle formation.

Hence it is feasible to assume the following mechanism of action: i) Linear peptide binds to the membrane, charge is involved in targeting (Fig. 7I); ii) formation of an α-helix structure by anchoring with Phe3 and Phe13 at the ester moieties of the lipids; iii) after reaching a threshold coverage, the α-helical peptides aggregate on the surface of the membrane into a loose bundle (Fig. 7II); iv) penetration of the aggregate into the top leaflet of the membrane; this increases the surface pressure of the top leaflet leading to “blistering” and eventual disintegration (Fig. 7III). Although the last stage of the mechanism has not been observed in simulations due to restrictions in the reachable time scales, it is reasonable to suggest that the “carpet” mechanism of action in case of Aurein 1.2 proceeds through aggregation induced membrane penetration, which is similar to the action of some transmembrane pore forming peptides such as alamethicin^63^. Hence our results point at an aggregation-driven membrane penetration mechanism as a common motif for amphiphilic helix-forming AMPs.

## Materials and Methods

Potassium dihydrogen phosphate (KH_2_PO_4_) and Potassium hydrogen phosphate (K_2_HPO_4_) were purchased from Fluka at ACS grade. Sodium chloride (NaCl) was purchased from Merck. Propan-2-ol, hydrogen peroxide (H_2_O_2_), and ammonia solution (28%, Analytical Univar Reagent) all were purchased from Sigma-Aldrich (Castel hill, NSW<Australia). 3-mercaptopropionic acid (MPA) (HPLC Grade, >99%) was purchased from Fluka, Bio- Chimia (Switzerland).Chloroform (ACS Reagent, 99.8%) was purchased from Sigma-Aldrich (Castel hill, NSW< Australia). Methanol was purchased from Sigma Aldrich (Methanol >99.9% A.C.S, Spectrophotometric grade) and ethanol was purchased from Sigma Aldrich (Ethanol, HPLC/ Spectrophotometric grade). Aurein 1.2 and Aurein 1.2 –COOH, 95% purity, were purchased from GLBiochem (China).

1,2-dimyristoyl-sn-glycero-3-phosphocholine(DMPC), sodium salt of 1,2-dimyristoyl-sn-glycero-3-phosphoglycerol (DMPG) and cholesterol were purchased from Avanti Polar Lipids (Alabaster, AL,USA). Lipids were dissolved in chloroform; in case of DMPG 3%methanol was added to improve solubility. Desired ratios of lipids: neat DMPC, DMPC/DMPG (4:1), (3:2), and DMPC/cholesterol (9:1) were measured into test tubes. The solvent was evaporated under a gentle stream of N_2_ and dried overnight. Liposomes were resuspended in 20 mM phosphate buffered saline (100 mM NaCl at pH 6.9).

### Quartz Crystal Microbalance with Dissipation Monitoring

Quartz Crystal Microbalance “with Dissipation Monitoring” (QCM) measurements were performed with a Q-SENSE E4 system (Q-Sense, Sweden). The sensor crystal used were 5 MHz, AT-cut, polished chips with evaporated gold sensor surface. The change of resonance frequency (Δf) and energy dissipation (ΔD) upon mass deposition were measured simultaneously at four different overtones of the fundamental frequency (where the fundamental frequency is the “first” overtone at 5 MHz). The (Δf) and (ΔD) values of the seventh overtone were presented. Sensor chips were rinsed with ethanol and dried under gentle stream of N_2_ gas, after that placed into a 3:1:1 mixture of ultrapure water 18.2 MΩ (Sartorius AG, Germany), hydrogen peroxide (30% solution) and ammonium hydroxide (28–30%solution) for 20 min at 70 °C. Then chips were rinsed with ultrapure water and dried. A clean gold surface was treated with 2% (w/w) MPA in Propan-2-ol overnight to form a self-assembled monolayer as a support for the biomimetic membrane^51^. The next day chips were rinsed in Propan-2-ol for 5 min to remove excess MPA and then assembled into the QCM chambers. At first water was injected into the chambers to hydrate the surface of the chips and then assay buffer was introduced. QCM experiments were repeated at least 3 times for each peptide and at each concentration. All experiments were performed by flushing 1 ml of liposome solution through the measurement chamber at 100 μL/min and at 19 °C^29^.

Peptides were injected into the QCM chamber once the formation of the single bilayer: 13–15 Hz frequency and 2.5–3.0 × 10^−6^ dissipation change was confirmed^51^. Control experiments on MPA coated gold confirmed the absence of peptide binding (10 μM Aurein 1.2; overall Δf = ~ – 1 Hz, ΔD = 0). Hence, all structural change measured correspond to the interaction between peptide and the membranes.

### QCM fingerprinting

Δf-ΔD plot can be used to provide an interaction fingerprint that is successfully used for the study of AMP-membrane interactions^29^,^57^. The Δf-ΔD curve correlates viscoelastic changes to mass uptake, which reflect changes in the membrane organization^29^. Thus, each direction in Δf-ΔD space reveals distinct mechanical changes. For the deposition or removal of thin viscoelastic layers, –Δf and ΔD are linearly proportional^56^. The direction [−f, +D] shows mass uptake; conversely if the interaction “vector” points towards [+f, −D], the process is mass loss^56^. Importantly this is different to the Sauerbrey approximation where dissipation is zero for all mass change^64^. Membranes deposited to SiO_2_ obey the Sauerbrey conditions; membranes deposited to MPA modified surface do not as the membrane is partially suspended^49^. “Stiffening”, i.e. increase in the shear modulus as suggested by McCubbin *et al*. would cause further negative frequency change; in the same article changes in the [+f, −D] direction were also interpreted as membrane removal^57^. Importantly, mass uptake or removal can be quantified from the Δf-ΔD plots.

In trendlines where ΔD is inversely proportional to –Δf, [−f, −D] and [+f, +D], the change predominantly occurs in viscoelastic properties, that is, distribution, density or mixing of the constituents of the deposit. These viscoelastic processes can be *qualitatively* assigned if informed by other experimental methods, computational results or by assessing possible mechanistic pathways. Importantly, Δf-ΔD fingerprints cannot be directly interpreted for the mechanism of interaction unless independent information is available about the interacting system. A full description of Δf-ΔD plot interpretation can be found in Refs. 29,65, 66, 67.

### Coarse grain simulations

Molecular dynamics simulations were carried out using the GROMACS package (vers. 4.6)^68^ and the coarse-grained MARTINI force field (vers. 2.2)^69^; using this representation, groups of three or four atoms are represented by just one interaction bead, reducing in this way the complexity of the system and allowing longer simulation timescales. Ionisable residues were assumed to be in their standard state at neutral pH. The non-amidated antimicrobial peptide had charged N and C termini; in the amidated case, the N terminus was kept as default whereas the C terminus was considered positively charged and with hydrogen donor properties. Other ionisable amino acids were considered to be in their standard state at neutral pH and chloride ions were included when necessary to keep the system neutrality.

All simulated systems consisted in a 10 × 10 × 8 nm simulation box that included a lipid bilayer and one or more peptides. In all cases the peptides started in the solvent and were allowed to move freely throughout the system. Therefore, neither binding nor aggregation was forced by the original setup.

The role of C-terminal amidation in binding was explored using one peptide (either amidated or non amidated) per simulation box and a lipid membrane composed of neat DMPC. The effect of the lipid composition in aggregation and binding was assessed in a different set of simulations where six peptides were placed in the simulation box. Both amidated and non amidated peptides were tested against a neat DMPC bilayer to provide further evidence about the role of amidation in the full process. For the other membranes (DMPC/Cholesterol, DMPC:DMPG (4:1) and DMPC:DMPG (3:2)) only wild type peptides were simulated.

Peptide, membrane and solvent were coupled separately to a thermal bath at 300 K Berendsen thermostat; a Parrinello-Rahman semi isotropic barostat at 1 bar was also included. The standard MARTINI scheme was followed to complete the system setup^69^. Each system was first minimised, then equilibrated for 5 ns and run without constraints for 1 microsecond. This last microsecond was used for analysis using standard GROMACS tools.

## Additional Information

**How to cite this article**: Shahmiri, M. *et al*. Controls and constrains of the membrane disrupting action of Aurein 1.2. *Sci. Rep*. **5**, 16378; doi: 10.1038/srep16378 (2015).

## Supplementary Material

Supplementary Material

## References

[b1] Gottler L. M. & Ramamoorthy A. Structure, membrane orientation, mechanism, and function of pexiganan—a highly potent antimicrobial peptide designed from magainin. Biochim. Biophys. Acta 1788, 1680–1686 (2009). 19010301 10.1016/j.bbamem.2008.10.009 PMC2726618

[b2] Brogden K. A. Antimicrobial peptides: pore formers or metabolic inhibitors in bacteria? Nat Rev Microbiol 3, 238–250 (2005). 15703760 10.1038/nrmicro1098

[b3] Pasupuleti M., Schmidtchen A. & Malmsten M. Antimicrobial peptides: key components of the innate immune system. Crit. Rev. Biotechnol. 32, 143–171 (2012). 22074402 10.3109/07388551.2011.594423

[b4] Andreu D. & Rivas L. Animal antimicrobial peptides: an overview. Biopolymers 47, 415–433 (1998). 10333735 10.1002/(SICI)1097-0282(1998)47:6<415::AID-BIP2>3.0.CO;2-D

[b5] García Olmedo F., Molina Fernández A., Alamillo J. M. & Rodriguez Palenzuela P. Plant defence peptides. Peptide Science 47, 479–491 (1998). 10333739 10.1002/(SICI)1097-0282(1998)47:6<479::AID-BIP6>3.0.CO;2-K

[b6] Ludtke S., He K. & Huang H. Membrane thinning caused by magainin 2. Biochemistry 34, 16764–16769 (1995). 8527451 10.1021/bi00051a026

[b7] Matsuzaki K., Sugishita K., Fujii N. & Miyajima K. Molecular basis for membrane selectivity of an antimicrobial peptide, magainin 2. Biochemistry 34, 3423–3429 (1995). 7533538 10.1021/bi00010a034

[b8] Tossi A., Sandri L. & Giangaspero A. Amphipathic, alpha-helical antimicrobial peptides. Biopolymers 55, 4–30 (2000). 10931439 10.1002/1097-0282(2000)55:1<4::AID-BIP30>3.0.CO;2-M

[b9] Ambroggio E. E., Separovic F., Bowie J. H., Fidelio G. D. & Bagatolli L. A. Direct visualization of membrane leakage induced by the antibiotic peptides: maculatin, citropin, and aurein. Biophys. J. 89, 1874–1881 (2005). 15994901 10.1529/biophysj.105.066589 PMC1366690

[b10] Maloy W. L. & Kari U. P. Structure–activity studies on magainins and other host defense peptides. Biopolymers 37, 105–122 (1995). 7893944 10.1002/bip.360370206

[b11] Huang H. W., Chen F.-Y. & Lee M.-T. Molecular mechanism of peptide-induced pores in membranes. Phys. Rev. Lett. 92, 198304 (2004). 15169456 10.1103/PhysRevLett.92.198304

[b12] Matsuzaki K. Magainins as paradigm for the mode of action of pore forming polypeptides. Biochim. Biophys. Acta 1376, 391–400 (1998). 9804997 10.1016/s0304-4157(98)00014-8

[b13] Shai Y. Mechanism of the binding, insertion and destabilization of phospholipid bilayer membranes by α-helical antimicrobial and cell non-selective membrane-lytic peptides. Biochim. Biophys. Acta 1462, 55–70 (1999). 10590302 10.1016/s0005-2736(99)00200-x

[b14] Yang L., Harroun T. A., Weiss T. M., Ding L. & Huang H. W. Barrel-stave model or toroidal model? A case study on melittin pores. Biophys. J. 81, 1475–1485 (2001). 11509361 10.1016/S0006-3495(01)75802-X PMC1301626

[b15] Shai Y. Mode of action of membrane active antimicrobial peptides. Peptide Science 66, 236–248 (2002). 12491537 10.1002/bip.10260

[b16] Shai Y. & Oren Z. From “carpet” mechanism to de-novo designed diastereomeric cell-selective antimicrobial peptides. Peptides 22, 1629–1641 (2001). 11587791 10.1016/s0196-9781(01)00498-3

[b17] Mani R., Buffy J. J., Waring A. J., Lehrer R. I. & Hong M. Solid-state NMR investigation of the selective disruption of lipid membranes by protegrin-1. Biochemistry 43, 13839–13848 (2004). 15504046 10.1021/bi048650t

[b18] Papo N. & Shai Y. Exploring peptide membrane interaction using surface plasmon resonance: differentiation between pore formation versus membrane disruption by lytic peptides. Biochemistry 42, 458–466 (2003). 12525173 10.1021/bi0267846

[b19] Giangaspero A., Sandri L. & Tossi A. Amphipathic α helical antimicrobial peptides. Eur. J. Biochem. 268, 5589–5600 (2001). 11683882 10.1046/j.1432-1033.2001.02494.x

[b20] Zelezetsky I. & Tossi A. Alpha-helical antimicrobial peptides—using a sequence template to guide structure–activity relationship studies. Biochim. Biophys. Acta 1758, 1436–1449 (2006). 16678118 10.1016/j.bbamem.2006.03.021

[b21] Apponyi M. A. . Host-defence peptides of Australian anurans: Structure, mechanism of action and evolutionary significance. Peptides 25, 1035–1054 (2004). 15203252 10.1016/j.peptides.2004.03.006

[b22] Epand R. F., Schmitt M. A., Gellman S. H. & Epand R. M. Role of membrane lipids in the mechanism of bacterial species selective toxicity by two α/β-antimicrobial peptides. Biochim. Biophys. Acta 1758, 1343–1350 (2006). 16564494 10.1016/j.bbamem.2006.01.018

[b23] Fink J., Merrifield R. B., Boman A. & Boman H. G. The chemical synthesis of cecropin D and an analog with enhanced antibacterial activity. J. Biol. Chem. 264, 6260–6267 (1989). 2495282

[b24] Matsuzaki K. . Modulation of magainin 2-lipid bilayer interactions by peptide charge. Biochemistry 36, 2104–2111 (1997). 9047309 10.1021/bi961870p

[b25] Bessalle R., Haas H., Goria A., Shalit I. & Fridkin M. Augmentation of the antibacterial activity of magainin by positive-charge chain extension. Antimicrob Agents Chemother 36, 313–317 (1992). 1605597 10.1128/aac.36.2.313 PMC188434

[b26] Tossi A., Scocchi M., Skerlavaj B. & Gennaro R. Identification and characterization of a primary antibacterial domain in CAP18, a lipopolysaccharide binding protein from rabbit leukocytes. FEBS Lett. 339, 108–112 (1994). 8313956 10.1016/0014-5793(94)80395-1

[b27] Scott M. G., Yan H. & Hancock R. E. Biological properties of structurally related alpha-helical cationic antimicrobial peptides. Infect Immun 67, 2005–2009 (1999). 10085049 10.1128/iai.67.4.2005-2009.1999 PMC96559

[b28] Dennison S. R. & Phoenix D. A. Influence of C-terminal amidation on the efficacy of modelin-5. Biochemistry 50, 1514–1523 (2011). 21241054 10.1021/bi101687t

[b29] Praporski S., Mechler A., Separovic F. & Martin L. L. Subtle Differences in Initial Membrane Interactions Underpin the Selectivity of Small Antimicrobial Peptides. ChemPlusChem 80, 91–96 (2015).

[b30] Hall K., Lee T.-H., Mechler A. I., Swann M. J. & Aguilar M.-I. Real-time Measurement of Membrane Conformational States Induced by Antimicrobial Peptides: Balance Between Recovery and Lysis. Sci. Rep. 4 (2014). 10.1038/srep05479 PMC4073255 24969959

[b31] Lohner K. & Prenner E. J. Differential scanning calorimetry and X-ray diffraction studies of the specificity of the interaction of antimicrobial peptides with membrane-mimetic systems. Biochim. Biophys. Acta 1462, 141–156 (1999). 10590306 10.1016/s0005-2736(99)00204-7

[b32] Boland M. P. & Separovic F. Membrane interactions of antimicrobial peptides from Australian tree frogs. Biochim. Biophys. Acta 1758, 1178–1183 (2006). 16580625 10.1016/j.bbamem.2006.02.010

[b33] White S. H. & Wimley W. C. Hydrophobic interactions of peptides with membrane interfaces. Biochim. Biophys. Acta 1376, 339–352 (1998). 9804985 10.1016/s0304-4157(98)00021-5

[b34] Shalev D. E., Mor A. & Kustanovich I. Structural consequences of carboxyamidation of dermaseptin S3. Biochemistry 41, 7312–7317 (2002). 12044162 10.1021/bi016013m

[b35] Moore A., Devine D. & Bibby M. Preliminary experimental anticancer activity of cecropins. Peptide Res. 7, 265–269 (1993). 7849420

[b36] Rozek T. . The antibiotic and anticancer active aurein peptides from the Australian Bell Frogs Litoria aurea and Litoria raniformis. Eur. J. Biochem. 267, 5330–5341 (2000). 10951191 10.1046/j.1432-1327.2000.01536.x

[b37] Lee T.-H. . Real-time quantitative analysis of lipid disordering by aurein 1.2 during membrane adsorption, destabilisation and lysis. Biochimica et Biophysica Acta 1798, 1977–1986 (2010). 20599687 10.1016/j.bbamem.2010.06.023

[b38] Seto G. W. J. . Interactions of the Australian tree frog antimicrobial peptides aurein 1.2, citropin 1.1 and maculatin 1.1 with lipid model membranes: Differential scanning calorimetric and Fourier transform infrared spectroscopic studies. Biochim. Biophys. Acta 1768, 2787–2800 (2007). 17825246 10.1016/j.bbamem.2007.07.018

[b39] Wang G. S., Li Y. F. & Li X. Correlation of three-dimensional structures with the antibacterial activity of a group of peptides designed based on a nontoxic bacterial membrane anchor. J. Biol. Chem. 280, 5803–5811 (2005). 15572363 10.1074/jbc.M410116200

[b40] Lee M.-T., Chen F.-Y. & Huang H. W. Energetics of pore formation induced by membrane active peptides. Biochemistry 43, 3590–3599 (2004). 15035629 10.1021/bi036153r

[b41] Lorenzon E. N., Sanches P. R. S., Nogueira L. G., Bauab T. M. & Cilli E. M. Dimerization of aurein 1.2: effects in structure, antimicrobial activity and aggregation of Candida albicans cells. Amino Acids 44, 1521–1528 (2013). 23519707 10.1007/s00726-013-1475-3

[b42] Mashaghi A., Swann M., Popplewell J., Textor M. & Reimhult E. Optical anisotropy of supported lipid structures probed by waveguide spectroscopy and its application to study of supported lipid bilayer formation kinetics. Anal. Chem. 80, 3666–3676 (2008). 18422336 10.1021/ac800027s

[b43] Fernandez D. I. . The antimicrobial peptide aurein 1.2 disrupts model membranes via the carpet mechanism. Phys Chem Chem Phys 14, 15739–15751 (2012). 23093307 10.1039/c2cp43099a

[b44] Mechler A. . Specific and Selective Peptide-Membrane Interactions Revealed Using Quartz Crystal Microbalance. Biophys. J. 93, 3907–3916 (2007). 17704161 10.1529/biophysj.107.116525 PMC2084233

[b45] Chen R. & Mark A. E. The effect of membrane curvature on the conformation of antimicrobial peptides: implications for binding and the mechanism of action. Eur. Biophys. J. 40, 545–553 (2011). 21267557 10.1007/s00249-011-0677-4 PMC3070085

[b46] Fernandez D. I., Sani M. A., Miles A. J., Wallace B. A. & Separovic F. Membrane defects enhance the interaction of antimicrobial peptides, aurein 1.2 versus caerin 1.1. Biochim. Biophys. Acta 1828, 1863–1872 (2013). 23506683 10.1016/j.bbamem.2013.03.010

[b47] Reimhult E., Hook F. & Kasemo B. Intact vesicle adsorption and supported biomembrane formation from vesicles in solution: Influence of surface chemistry, vesicle size, temperature, and osmotic pressure. Langmuir 19, 1681–1691 (2003).

[b48] Keller C. A. & Kasemo B. Surface specific kinetics of lipid vesicle adsorption measured with a quartz crystal microbalance. Biophys. J. 75, 1397–1402 (1998). 9726940 10.1016/S0006-3495(98)74057-3 PMC1299813

[b49] Hasan I. Y. & Mechler A. Viscoelastic changes measured in partially suspended single bilayer membranes. Soft Matter 11, 5571–5579 (2015). 26073288 10.1039/c5sm00278h

[b50] Tayebi L. . Long-range interlayer alignment of intralayer domains in stacked lipid bilayers. Nature Materials 11, 1074–1080 (2012). 23085566 10.1038/nmat3451

[b51] Mechler A. . Structure and homogeneity of pseudo-physiological phospholipid bilayers and their deposition characteristics on carboxylic acid terminated self-assembled monolayers. Biomaterials 30, 682–689 (2009). 19000635 10.1016/j.biomaterials.2008.10.016

[b52] Gehman J. D. . Effect of Antimicrobial Peptides from Australian Tree Frogs on Anionic Phospholipid Membranes. Biochemistry 47, 8557–8565 (2008). 18652483 10.1021/bi800320v

[b53] Wadhwani P. . Membrane-active peptides and the clustering of anionic lipids. Biophys. J. 103, 265–274 (2012). 22853904 10.1016/j.bpj.2012.06.004 PMC3400765

[b54] Oren Z. & Shai Y. Mode of action of linear amphipathic alpha-helical antimicrobial peptides. Biopolymers 47, 451–463 (1998). 10333737 10.1002/(SICI)1097-0282(1998)47:6<451::AID-BIP4>3.0.CO;2-F

[b55] Dennison S. R., Morton L. H. G. & Phoenix D. A. Effect of Amidation on the Antimicrobial Peptide Aurein 2.5 from Australian Southern Bell Frogs. Protein and Peptide Letters 19, 586–591 (2012). 22519529 10.2174/092986612800494110

[b56] Voinova M. V., Rodahl M., Jonson M. & Kasemo B. Viscoelastic acoustic response of layered polymer films at fluid-solid interfaces: continuum mechanics approach. Phys. Scr. 59, 391 (1999).

[b57] McCubbin G. . QCM-D fingerprinting of membrane-active peptides. Eur. Biophys. J. 40, 437–446 (2011). 21161523 10.1007/s00249-010-0652-5

[b58] Mechler A. . Specific and selective peptide-membrane interactions revealed using quartz crystal microbalance. Biophys. J. 93, 3907–3916 (2007). 17704161 10.1529/biophysj.107.116525 PMC2084233

[b59] Lorenzón E. N., Piccoli J. P. & Cilli E. M. Interaction between the antimicrobial peptide Aurein 1.2 dimer and mannans. Amino Acids 46, 2627–2631 (2014). 25209238 10.1007/s00726-014-1832-x

[b60] Sood R. & Kinnunen P. K. J. Cholesterol, lanosterol, and ergosterol attenuate the membrane association of LL-37(W27F) and temporin L. Biochim. Biophys. Acta 1778, 1460–1466 (2008). 18358828 10.1016/j.bbamem.2008.02.014

[b61] McMullen T. P. W., Lewis R. N. A. H. & McElhaney R. N. Cholesterol–phospholipid interactions, the liquid-ordered phase and lipid rafts in model and biological membranes. Curr. Opin. Colloid Interface Sci. 8, 459–468 (2004).

[b62] Sani M. A., Gagne E., Gehman J. D., Whitwell T. C. & Separovic F. Dye-release assay for investigation of antimicrobial peptide activity in a competitive lipid environment. European Biophysics Journal with Biophysics Letters 43, 445–450 (2014). 24906225 10.1007/s00249-014-0970-0

[b63] Pieta P., Mirza J. & Lipkowski J. Direct visualization of the alamethicin pore formed in a planar phospholipid matrix. Proceedings of the National Academy of Sciences of the United States of America 109, 21223–21227 (2012). 23236158 10.1073/pnas.1201559110 PMC3535624

[b64] Sauerbrey G. & Jung G. Vibrational Modes of Planoconvex Quartz Plates. Zeitschrift Fur Angewandte Physik 24, 100-& (1968).

[b65] Knappe D. . Oncocin (VDKPPYLPRPRPPRRIYNR-NH2): A Novel Antibacterial Peptide Optimized against Gram-Negative Human Pathogens. J. Med. Chem. 53, 5240–5247 (2010). 20565063 10.1021/jm100378b

[b66] McCubbin G. A. . QCM-D fingerprinting of membrane-active peptides. European Biophysics Journal with Biophysics Letters 40, 437–446 (2011). 21161523 10.1007/s00249-010-0652-5

[b67] Piantavigna S. . Cell Penetrating Apidaecin Peptide Interactions with Biomimetic Phospholipid Membranes. Int. J. Pept. Res. Ther. 15, 139–146 (2009).

[b68] Pronk S. . GROMACS 4.5: a high-throughput and highly parallel open source molecular simulation toolkit. Bioinformatics 29, 845–854 (2013). 23407358 10.1093/bioinformatics/btt055 PMC3605599

[b69] de Jong D. H. . Improved Parameters for the Martini Coarse-Grained Protein Force Field. Journal of Chemical Theory and Computation 9, 687–697 (2013). 10.1021/ct300646g 26589065

